# Effect on the expression of *drd2* and *drd3* after neonatal lesion in the lymphocytes, nucleus accumbens, hippocampus and prefrontal cortex: comparative analysis between juvenile and adult Wistar rats

**DOI:** 10.1186/s41065-016-0018-9

**Published:** 2016-11-22

**Authors:** Alma Delia Genis-Mendoza, Carlos Alfonso Tovilla-Zárate, Lilia López-Narvaez, Patricia Mendoza-Lorenzo, Patricia Ostrosky-Wegman, Humberto Nicolini, Thelma Beatriz González-Castro, Yazmin Hernández-Diaz

**Affiliations:** 1 Instituto Nacional de Medicina Genómica (INMEGEN), Servicios de Atención Psiquiátrica (SAP), Secretaria de Salud, D.F. Mexico, Mexico; 2 División Académica Multidisciplinaria de Comalcalco, Universidad Juárez Autónoma de Tabasco, Comalcalco, Tabasco Mexico; 3 Hospital General de Yajalón, Secretaria de Salud. Yajalón, Chiapas, Mexico; 4 Instituto de Investigaciones Biomédicas, UNAM, D.F, Mexico, Mexico; 5 División Académica Multidisciplinaria de Jalpa de Méndez, Universidad Juárez Autónoma de Tabasco, Jalpa de Méndez, Tabasco Mexico; 6 Carracci Medical Group, Carracci 107. Insurgentes Extremadura, Ciudad de México, D.F. 13740 Mexico

**Keywords:** NLVH, Peripheral lymphocyte, Dopamine expression

## Abstract

**Background:**

Neonatal lesion in the ventral hippocampus (NLVH) is a validated animal model to study schizophrenia from a neurodevelopmental perspective. This animal model is also used to investigate how neonatal lesions may alter the genetic expression of dopaminergic receptors. The present study compares mRNA expression levels of dopamine receptors (*drd2* and *drd3*) in lymphocytes and brain of NLVH animals at two different age stages: young and adult.

**Methods:**

The NLVH procedure was performed on 20 male Wistar rats at postnatal days 5–7. The mRNA expression levels of *drd2* and *drd3* genes in lymphocytes, nucleus accumbens, hippocampus and prefrontal cortex were measured and analyzed at postnatal days 45 and 90. The results were compared and contrasted with respective sham groups.

**Results:**

In lymphocytes, only in NLVH-adult group we observed *drd2* mRNA expression, while *drd2* mRNA expression was not observed in the NLVH-juvenile rats; on the other hand, the *drd3* mRNA expression did not show significant statistical differences. In hippocampus no differences were observed between *drd2* mRNA or *drd3* mRNA expression when comparing juvenile/adult shams with NLVH groups. In the prefrontal area, a decrease in *drd2* mRNA expression levels were observed in the NLVH-adult group (F(1,3) = 52.83, *p* = 0,005) in comparison to the sham-adult group. Finally, in the nucleus accumbens, a strong decrease of *drd3* mRNA expression was observed in the NLVH-adult group in comparison to the sham-adult group (F(1,3) = 123,2, *p* < 0.001).

**Conclusions:**

Our results show that differences in *drd2* and *drd3* mRNA levels in NLVH-adults are patent when compared to the sham-adult group or with the NLVH-juvenile group. These findings suggest that the expression levels may be regulated during adulthood, leading to behavioral and neurochemical changes related to schizophrenia. Therefore, more studies are necessary to determine the role of dopamine receptors as possible molecular markers for neurodevelopmental changes associated with schizophrenia.

## Background

Schizophrenia is a mental illness which causes a heavy economic burden to society; it affects 1 % of the world population, and it usually develops during the reproductive stage of the person and remains present throughout his/her life [1, 2]. The difficulties to obtain adequate human brain-samples and the increasing necessity to study schizophrenia have led to the development of several animal models. These animal models can be divided into three categories: pharmacological models, genetic models (induced genetic mutations or deletions) and neurodevelopmental models (induced by a natural or neurotoxic injury or by environmental factors during neurodevelopment) [3–5].

The neonatal lesion in the ventral hippocampus (NLVH) model in animals is based on a neurodevelopmental hypothesis implicating that at an early age (probably during the prenatal stage) fundamental alterations may induce the occurrence of schizophrenia [6–8]. For instance, Tseng et al. (2009) suggest that NLVH disrupts the dorso-lateral cortical morphogenesis, since these authors observed that the dopaminergic pathway sets off from the ventral hippocampus to the dorso-lateral area in rat embryos [9]. This effect is more evident in young rats whereas during adolescence rats lose the *drd2* modulation in cortical interneurons, as well as the onset of cortical maturation [10]. These findings increase the possibility that maturation of local inhibitory circuits within the PFC may be caused by NLVH [11]. In mental diseases, particularly those treated with antipsychotic medication (including schizophrenia), dopaminergic receptors are of particular interest [3, 4]. The gene expression of dopamine receptors has been directly studied in the brain, and it has also been monitored in other cell populations such as lymphocytes [12]. Therefore, peripheral dopamine receptors may serve as representative markers for changes occurring within the central nervous system associated with neuropsychiatric syndromes such as depression and schizophrenia [13].

There are various hypotheses trying to explain the impairment in dopamine transmission [14]. This monoamine dysfunction may be due to immune-inflammatory or neuroendocrine causes or to neuroplasticity malfunctioning [15]. Some authors ascertain that dopamine can directly affect the immune system. They suggest that further research on animal models must address not only the effects of dopaminergic substances on peripheral expression of dopamine receptors, but also the correlation between the regulation of central and peripheral dopamine receptors [16, 17]. To explore this possibility the present study compares mRNA expression of dopamine receptors (*drd2* and *drd3*) in lymphocytes and in some areas of rat brain with NLVH at two different age periods: young and adult.

## Methods

### Animals

The cohort of animals in this report is the same used in a previous study [18]. Twenty male Wistar rats were used in this study; all animals came from pregnant rats in our laboratory. They were individually housed in breeding cages under a 12-h light/12-h dark cycle. The sample was divided into four groups: 1) NLVH-juvenile group, 2) sham-juvenile group, 3) NLVH-adult group and 4) sham-adult group. Each group was housed in a cage and kept under an inverted 12-h light/12-h dark cycle (lights off at 10:00 a.m.) and controlled temperature (22.2 °C). Testing was routinely performed between 10:00 a.m. and 3:00 p.m.; animals had free access to food and water throughout the experiment [5]. The local committee of ethics on animal experimentation approved the experimental procedures, which complied with the regulations established in the Mexican official norm for the use and care of laboratory animals, “NOM-062-ZOO-1999”, as well as the regulations of the Ethics Committee of the International Association for the Study of Pain [19].

### Neonatal ventral hippocampus lesion

Bilateral NLVH was performed in neonatal specimens as described by Lipska et al. [20]. Litters of four to eight male pups were grouped and submitted to surgical alteration on postnatal days five to seven (PD 5–7) (weight 10–13 g). Pups were randomly assigned to lesion or sham status and anesthetized with hypothermia by placing the animals on ice for 10–15 min. Pups were then taped to a platform fixed to a stereotaxic Kopf instrument and skin incisions overlying the skull were made. Bilateral ventral hippocampus lesions were performed by infusing 0.15 μl/min (over a period of 2 min) of ibotenic acid (Sigma, St Louis, MO, USA) (lesion) or PBS solution (for the sham groups) through a Hamilton needle in the following coordinates relative to Bregma: anterior-posterior (AP) -2.0; medium lateral (ML) ±2.5, and dorsum-ventral (DV) -3.3 in relation to Bregma [21]. We had successfully established the rat NLVH model as reported elsewhere [18].

### RNA extraction

The animals were sacrificed by rapid decapitation at 45 and 95 days old. Following this procedure, their brains were dissected and the following regions were selected: prefrontal cortex, nucleus accumbens and hippocampus. Trunk blood was collected and lymphocytes were isolated by differential centrifugation using a Ficoll density gradient centrifugation and stored at −70 °C until use. Subsequently, RNA extraction for each brain region was performed following the Trizol-method (Life Technologies) according to the manufacturer’s instructions. The RNA obtained was quantified by plaque fluorometry utilizing fluorescent dyes to determine the concentration of nucleic acids in a simple using the Quant-IT RiboGreen RNA assay (Invitrogen) according to the manufacturer’s instructions, as well as a Fluoroskan Ascent equipment (Thermo Electron Corporation, Vantaa, Finland). The cDNA was synthesized using Super Script III First-Strand Synthesis Supermix (Invitrogen) according to the manufacturer’s instructions. The quantification of *drd2* and *drd3* expression was performed through TaqMan Gene Expression Assays using the Applied Biosystems 7900HT Fast Real-Time PCR Systems. Relative expression was evaluated through 18S rRNA gene as the endogenous control. The comparative C_T_ method (2∆∆CT) was used to calculate *drd2* and *drd3* expression levels in the brain samples and lymphocytes.

### *drd2* and *drd3 e*xpression assays

Gene expression of *drd2* and *drd3* was determined by real time PCR, running the cDNA reactions mixtures on a 4900 Real Time PCR System (Applied Biosystems). The initial cDNA quantity was 25 ng, although increasing concentrations were also used (50, 100 and 200 ng). We utilized TaqMan® rat probes *drd2*-10 FAM (Rn00561126-m1) and *drd3* FAM (Rn00691132-m1) (Applied Biosystems), in multiple assays (by triplicate), using ribosomal RNA as endogenous gene (TaqMan Ribosomal RNA Control Reagents VIC® dye; Applied Biosystems). Data were analyzed with the C_T_ comparative method (2ΔΔCT). All the experiments were run in triplicate. Ventral hippocampus lesions were verified by means of histological stained sections for both juvenile and adult stages.

### Statistical analysis

Data are presented as mean ± SEM. For each region: lymphocytes, hippocampus, prefrontal area and nucleus accumbens a Two-Way ANOVAs was performed to compare the sham and the NLVH groups. To compare juveniles against adults, a two-way ANOVA was performed followed by Bonferroni test for post hoc. The level of statistical significance was established at *p* < 0.05.

## Results

### *drd2* and *drd3* mRNA expression levels in lymphocytes of juvenile and adult groups

**Fig. 1:**
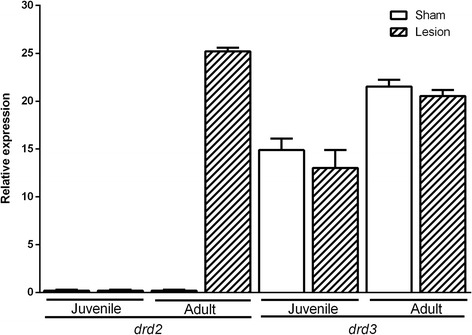
*drd2* and *drd3* gene expression in lymphocytes of juvenile and adult groups. ***p < 0.001

Gene expression and quantification of *drd2* mRNA in lymphocytes were higher in NLVH-adult rats than in the sham-adults (where no *drd2* mRNA expression was observed). This means that the expression of this marker was only present when the lesion procedure was performed (Fig. 1). In the sham-adult group, only the endogenous expression of the 18S rRNA gene was detected. In the juvenile group no expression was observed. In consequence, the expression of the *drd2* mRNA in the NLVH-group adults showed major expression that in NLVH-juvenile groups.

In contrast, *drd3* mRNA expression levels were observed in both groups. However, no differences were observed when comparing the *drd3* mRNA expression between sham-adults and NLVH-adults (F (1,4) =3.85, *p* = 0.12) (Fig. 1). Equally, no differences were observed between the juvenile groups (F (1,4) =4.15, *p* = 0.38). However, the expression of *drd3* mRNA in the sham-adult group was higher than in the sham-juvenile group (mean diff. -6.62 CI diff:-9.01- -4.24, *p* < 0.05). Equally the expression of NLVH-adults showed higher expression than the NLVH-juvenile group (mean diff. -7.51 CI diff:-9.87- -5.12, *p* < 0.05).

### drd2 and drd3 mRNA expression levels in the hippocampus of juvenile and adult groups

The *drd2* mRNA expression levels (F (3,13) = 3.93, *p* = 0.06) in the hippocampus comparing NLVH-adults and sham-adults were no different (F (1,4) = 3.76, *p* = 0.12); likewise, the comparison between NLVH-juveniles and sham-juveniles showed no difference of *drd2* mRNA expression levels (F (1,4) = 3.29, *p* = 0.14). When we compared the expression of *drd2* mRNA between sham-adults and sham-juveniles not statistical differences were observed (mean diff. -3.05 CI diff:-6.69- 0.59, *p* > 0.05), or when comparing NLVH-adults and NLV-juveniles (mean diff. 1.60 CI diff:-2.04- 5.24, *p* > 0.05).

**Fig. 2:**
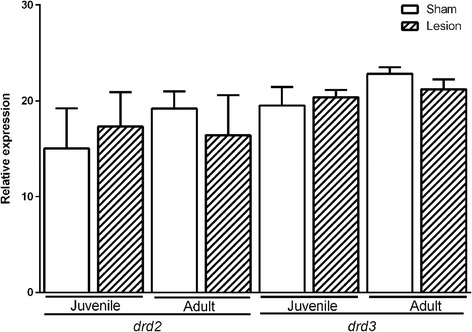
*drd2* and *drd3* gene expression in the hippocampus of juvenile and adult groups. Two-Way ANOVAs test

In addition, no differences of the *drd3* mRNA expression levels were found between sham-adults and NLVH-adults (F (1,4) = 5.72, *p* = 0.07) (Fig. 2). Similarly, no differences in *drd3* mRNA expression levels were observed between sham-juveniles and NLVH-juveniles (F (1,4) = 1.11, *p* = 0,33). The expression of *drd3* mRNA in the sham-adult group showed no statistical difference when compared with the sham-juvenile group (mean diff. -3.50 CI diff:-6.69- 0.59, *p* > 0.05); equally, the comparison between NLVH-juvenile and NLVH-adults showed no difference of *drd3* mRNA expression (mean diff. 1.60 CI diff:-2.04- 5.24, *p* > 0.05).

### drd2 and drd3 mRNA expression levels in prefrontal area of juvenile and adult groups

**Fig. 3:**
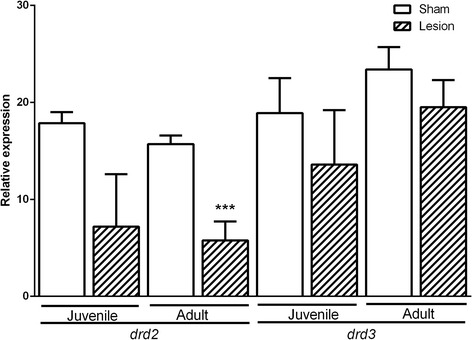
*drd2* and *drd3* gene expression in prefrontal cortex of juvenile and adult groups. Two-Way ANOVAs test. ***p < 0.001

In the prefrontal cortex (Fig. 3), a difference was observed when comparing the adults, as the sham group showed higher levels of *drd2* mRNA expression than the NLVH group (F(1,3) = 52.83, *p* = 0.005). However, no differences of *drd2* mRNA expression levels were observed in sham-juveniles when compared with the NLVH-juveniles (F(1,3) = 5,5, *p* = 0.1). When a comparison between juvenile and adult groups was performed, we did not observe differences between the NLVH groups (mean diff. 6.53 CI diff:-1.12- -14.19, *p* > 0.05) or between the sham groups (mean diff. 2.64 CI diff:-4.96 – 10.35, *p* > 0.05).

Regarding the *drd3* mRNA expression levels, no differences were observed between the sham-adults and NLVH-adults (F (1,3) = 2.30, *p* = 0.22). Similarly, no differences in *drd3* mRNA expression levels were observed between sham-juveniles and NLVH-juveniles (F (1,3) = 1.51, *p* = 0.30) (Fig. 3). In addition, no differences of the *drd3* mRNA expression levels were found between the NLVH-juveniles and NLVH-adults (mean diff. 7.19 CI diff:-19.01 – 4.61, *p* > 0.05) (Fig. 3). Also, no differences of the *drd3* mRNA expression levels were observed between sham-juveniles and sham-adults (mean diff. -4.75 CI diff:-16.56 – 7.06, *p* > 0.05).

### drd2 and drd3 mRNA expression levels in the nucleus accumbens of juvenile and adult groups

**Fig. 4:**
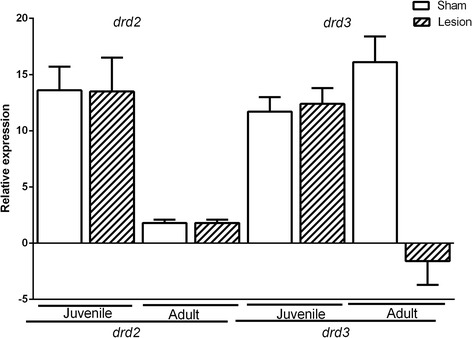
*drd2* and *drd3* gene expression in nucleus accumbens of juvenile and adult groups. Two-Way ANOVAs test. *p* < 0.001

With respect to the nucleus accumbens, when the expression levels of *drd2* mRNA were evaluated in NLVH-adults, no difference was observed when compared with the sham-adults (F (1,3) =5,5, *p* = 0.1). Similarly, no statistical differences were observed when the sham-juveniles and NLVH-juveniles were compared (F (1,3) = 0.01, *p* = 0.90). However, in the NLVH-adult group a marked decrease of the *drd2* expression levels was detected in comparison with the NLVH-juvenile group (mean diff. 12.09 CI diff:7.68 – 16.50, *p* < 0.05); equally, the expression in the sham-adult group was smaller than in the sham-juvenile group (mean diff. 11.87 CI diff:7.46 – 16.28, *p* < 0.05) (Fig. 4).

When the drd3 mRNA expression levels were evaluated in adults, a marked decrease of *drd3* mRNA was observed in NLVH-adults when compared with sham-adults (F (1,3) = 123.2, *p* < 0.001) (Fig. 4). Between the juvenile groups no differences were observed (F (1,3) = 0.074, *p* = 0.45). Finally, the expression of *drd3* mRNA in the sham-adult group was higher than in the sham-juvenile group (mean diff. -5.25, *p* < 0.05); whereas the comparison between NLVH-juveniles and NLVH-adults showed a lower expression of drd3 in the adult group (mean diff. 12.48, *p* < 0.01).

## Discussion

The present study provides important evidence about the role of *drd2* and *drd3* genes in lymphocytes and in some brain areas (hippocampus, prefrontal cortex and nucleus accumbens) in NLVH rats at two different age periods: young and adult. Our study showed that the *drd3* mRNA expression levels in lymphocytes were detected from postnatal day 45. In contrast, *drd2* expression was only detected in the NLVH-adult group (90 days of age); which may indicate late neurodevelopmental effects. In rats, synaptic pruning in *drd1* and *drd2* receptors occurs during the peri-adolescent period (postnatal days 42–44). It has been described that after this pruning, the onset of schizophrenia commonly occurs [22, 23]. In the juvenile stage no expression changes were found in any of the tissues studied. Previously, Flores and colleagues conducted a study of dopamine receptors expression using the NLHV model and autoradiography at 41 days of age and found no changes in the expression of *drd1*, *drd2* and *drd3* receptors [24]. El-Rawas et al. also evaluated *drd2* expression by immunohistochemistry in several brain regions and only encountered changes in the putamen [25]. In our evaluation, only changes in adult rats were detected (Figs. 1, 3 and 4). These findings suggest that the expression levels of some dopamine receptors are regulated during adulthood, leading to behavioral and neurochemical changes related to schizophrenia.

Regarding the group of NLHV-adult rats, a decreasing trend for *drd3* and a clear decrease in *drd2* mRNA expression was observed in the prefrontal cortex compared to sham-adults (Fig. 3). Flores et al. used autoradiography and reported no changes in expression observed in this molecule in animals at 62 days of age [24, 26]. Possibly, the *drd2* expression may be observed at later ages, since we detected these differences only after 90 days. The regulation of *drd2* expression in the prefrontal cortex of the adult rats group may indicate late neurodevelopmental effects. Cognitive deficits in humans have been correlated with an imbalance of D1 and D2 receptors in the prefrontal cortex [27, 28]. The prefrontal cortex, hippocampus and the developmentally dependent synaptic pruning in the juvenile stage may contribute to the present results [22]. In rats, neuronal pruning of *drd1* and *drd2* receptors occurs during the peri-adolescent period (42–44 days). In humans, after this pruning takes place, it is possible to observe the symptoms of schizophrenia [22, 23]. Furthermore, the development of the prefrontal cortex in the early gestation stage and in adolescence represent two critical periods of vulnerability for schizophrenia in humans [29].

In the case of the mRNA expression in the hippocampus, no changes were observed for *drd2* or *drd3*. However, with regard to the accumbens core, a clear decrease in the *drd2* transcript was observed in adults against juveniles (Fig. 4), possibly due to neuronal pruning, since the dopamine hypothesis suggests a disturbed and hyperactive dopaminergic signal transduction; the evidence indicates that dopamine receptors are produced in excess before puberty (day 40). Moreover, Teicher et al. reported that at 40 days of age there is a slight decrease in *drd2* receptors in the nucleus accumbens [22]. Also, a decrease of the*drd3* expression was observed in the NLVH-adults, as reported by Flores et al. when they analyzed rats at 63 days of age using autoradiography [24]. Several reports of reduced *drd3* mRNA in schizophrenic brains further support the potential implication of this receptor subtype in this disorder [30, 31].

We consider that when our rats reached adulthood, the *drd2* was expressed only as a response to the lesion performed when neonates. In addition, we observed a reduction of the *drd2* expression in the prefrontal cortex in NLVH-adult rats. Conversely, in lymphocytes we encountered high *drd2* expression levels also in NLVH-adults. These preliminary results appear contradictory, which may indicate that blood changes do not reflect brain changes in the expression of the message for this receptor. Therefore, it is important to further investigate expression profiles in other brain areas represented in schizophrenia. On the other hand, it was not possible to detect *drd2* expression in the sham groups; this is consistent with other reports, which indicates that although all five dopaminergic receptors are present in lymphocytes of peripheral blood, the *drd2* expression is not observed in healthy animals without NLVH manipulation (sham groups) [16]. Therefore, *drd2* expression may be directly related to NLVH in this study.

To date, there are few reports on dopaminergic receptors using the NLVH model. Using microarray technology, *drd2* over-expression has been observed in patients; hence this dopamine receptor has been proposed as a peripheral blood biomarker [32, 33]. Another study showed that *drd2* mRNA levels in lymphocytes of peripheral blood were correlated with positive symptoms in acute schizophrenia patients [34]. The analysis of dopamine receptors in lymphocytes of peripheral blood could be a useful tool for assessing the properties of dopaminergic function in psychopathological traits of schizophrenia [35, 36]. Finally, further studies that allow the analysis of behavioral alterations in rats are needed to confirm the findings of this study.

## Conclusions

We encountered significant differences in the effect of NLVH on *drd2* and *drd3* mRNA expression levels among the brain regions analyzed in rat adulthood. These findings suggest that expression levels of some dopamine receptors may be regulated during adulthood, leading to behavioral and neurochemical changes associated with schizophrenia. However, more studies are necessary to establish whether changes in *drd2* mRNA expression levels could represent a molecular marker for neurodevelopmental changes related to schizophrenia.
